# Cu-Containing Faujasite-Type Zeolite as an Additive in Eco-Friendly Energetic Materials

**DOI:** 10.3390/molecules29133184

**Published:** 2024-07-04

**Authors:** Łukasz Kuterasiński, Marta Sadowska, Paulina Żeliszewska, Bogna Daria Napruszewska, Małgorzata Ruggiero-Mikołajczyk, Mateusz Pytlik, Andrzej Biessikirski

**Affiliations:** 1 Jerzy Haber Institute of Catalysis and Surface Chemistry, Polish Academy of Sciences, ul. Niezapominajek 8, 30-239 Kraków, Poland; marta.sadowska@ikifp.edu.pl (M.S.); paulina.zeliszewska@ikifp.edu.pl (P.Ż.); bogna.napruszewska@ikifp.edu.pl (B.D.N.); malgorzata.ruggiero-mikolajczyk@ikifp.edu.pl (M.R.-M.); 2 Central Mining Institute-National Research Institute, 1 Gwarków Square, 40-166 Katowice, Poland; mpytlik@gig.eu; 3 Faculty of Civil Engineering and Resource Management, AGH University of Krakow, Al. Mickiewicza 30, 30-059 Kraków, Poland

**Keywords:** Cu-faujasite, modifier, ANFO, energetic properties

## Abstract

Regarding the current state of the art on the utilization of zeolites in industry, the application of zeolites as an additive to eco-friendly energetic materials indicates the innovative character of the present research. One of the most commonly used energetic materials in the mining industry (engineering works) is ANFO (ammonium nitrate fuel oil), due to its easy and cheap production procedure as well as its good energetic properties and vast possibilities for modification. In the present research, we investigated Cu-zeolite with a faujasite structure (Cu-FAU) as a modifier of ANFO-based energetic materials. Analysis of the results obtained from thermodynamic calculations of energetic performance led to the conclusion that the application of Cu-faujasite as an additive to ANFO resulted in a relevant reduction in the total emission of post-decomposition fumes, with simultaneous enhancement of the energetic properties of the energetic material, which corresponded with the changes in the status of the surface and the reduced thermal effect accompanying the ammonium nitrate’s decomposition. From analysis of both the energetic performance and fumes, it may be concluded that our eco-friendly and enhanced energetic material can be used as a low-emission source of energy for the quarrying of raw materials.

## 1. Introduction

Zeolites are 3D crystalline and microporous aluminosilicate materials. As microporous solids, zeolites are characterized by many unique properties, which are responsible for their high acidity, shape selectivity, surface area, and thermal stability. Due to the presence of acid-active sites, this group of aluminosilicates can be also used in many catalytic industrial processes, including cracking, esterification, alkylation, isomerization, the production of fine chemicals, and environmental protection [1,2]. Zeolites can be used in many industrial, acid-catalyzed reactions, e.g., methanol-to-olefins (MTO) conversion [3,4], methanol-to-gasoline (MTG) conversion [5,6], ethylbenzene dealkylation [7], xylene isomerization [8,9], benzene alkylation [10], propylene oligomerization [11], n-alkane cracking [12,13,14], toluene disproportionation [15,16], methanol’s conversion to dimethyl ether [17], esterification of fatty acids [18,19], dewaxing [20], the production of fine chemicals [21], and many other reactions.

Other non-catalytic applications of zeolites include water purification and softening [22], advanced nuclear reprocessing methods [23], the separation of gases [24], thermochemical storage of solar heat harvested from solar thermal collectors [25], building materials [26], agriculture [27], and many other applications.

Analysis of the literature on the application of zeolites in industry leads to the conclusion that the application of zeolites as modifiers of energetic materials constitutes an innovative characteristic of the present research. One of the most commonly used energetic materials in civil applications (mainly in open-pit mining or macronivelations) is ANFO, due to its easy and cheap production procedure as well as its good energetic properties [28]. In turn, the particular attractiveness of zeolites as modifiers of ANFO-based energetic materials implies their chemical composition. For instance, the presence of Al, an organic template, or even introduced metallic guest atoms in extraframework positions in zeolite can play the role of combustible components, as well as sensitizers (similar to fuel oil), and can lead to the modification of energetic effects such as pressure, temperature, heat, strength, and the velocity of energetic decomposition [29,30,31,32,33]. In turn, the presence of Si (connected with O) in zeolites could be treated as an inert material, which can absorb some of the energy and, therefore, decrease the heat and strength of the energetic decomposition, among other factors [34,35,36]. Independent of the character of the additive (an inert material or substance that is chemically active), its addition will result in an influence on the material density and oxygen balance, which will result in changes in energetic properties: energy, heat, and pressure of decomposition, which, in turn, affect the composition of the fumes. At this point, it must be noted that combustible components for creating ANFO-based energetic materials constitute not only organic additives (like fuel oil, activated carbon, charcoal, hydrocarbons and their derivatives, etc.) but also inorganic ingredients (like metallic dust, salts, oxides, and so on) [29,30,31,32,33]. The co-existence of elements in zeolites that can play the role of either combustible components (organic templates, Al, and/or other guest metals) or inert phases (Si-O groups) makes zeolites particularly attractive dual-use modifiers in the industry of energetic materials. 

Due to the commonly known application of Cu-zeolites as DeNO_x_ catalysts [37,38,39,40,41,42,43,44,45], we decided to implement zeolites containing copper as possible ANFO additives for reducing the toxic post-decomposition fumes produced during the decomposition reaction of ANFO-based energetic materials. Within the conducted research, we also tried to find an answer to the question of whether the form of copper species could determine the amount and/or chemical composition of post-decomposition fumes produced in the presence of Cu-zeolites.

In our previous paper [46], we reported that the way of preparing Cu-faujasite of Si/Al = 31 (FAU31) determined the status and properties of Cu sites in these samples. We indicated that Cu was found as a monovalent cation existing both in exchange extraframework positions (Cu^+^_exch_) and as oxides (Cu^+^_ox_). Copper can also form divalent species (Cu^2+^_exch_ and CuO). The chemical form of Cu depended on the copper content in zeolite FAU31 and on the form of FAU31 support to which the Cu was introduced: faujasite in protonic form (HFAU31), or FAU31 in sodium form as a result of ion exchange of HFAU31 with NaNO_3_ (NaFAU31). The CuHFAU31 systems obtained from the protonic form of faujasite contained mainly Cu^+^_exch_, whereas the CuNaFAU31 counterparts derived from the sodium form of faujasite were characterized by much higher amounts of Cu^+^_ox_ and Cu^2+^. 

We also determined the reducibility (defined as susceptibility to reduction by hydrogen) of specific chemical forms of copper in FAU31 zeolite, with simultaneous monitoring of the Cu’s chemical form using NO and CO sorption as probe molecules [47]. We found that Cu species were prone to reduction in the following sequence: Cu^+^_oxide_ > Cu^2+^_exch_. > Cu^2+^_oxide_ > Cu^+^_exch_. We also observed the production of Brønsted acid sites, as a result of the reduction of copper species with hydrogen according to the following reactions: 2Cu^+^ + H_2_= 2Cu^0^ + 2H^+^ and Cu^2+^ + H_2_= Cu^0^ + 2H^+^. 

In the present paper, we report the synthesis of ANFO consisting of ammonium nitrate (AN), fuel oil (FO), and the Cu-containing zeolite FAU31 acting as a modifier. The zeolite was added to the ANFO via a simple mixing procedure. The weight loading of the zeolite in the resulting ANFO-based material reached 5% wt. Copper was introduced into the zeolite via a wet impregnation procedure and in various chemical forms. Separate studies were performed for the reference ANFO sample, which was deprived of additive zeolite. The prepared ANFO samples underwent physicochemical characterization, including crystallinity, structure, the status of the surface, thermal properties, and thermodynamic calculations concerning energetic properties (including the velocity, temperature, pressure, heat, and the analysis of post-decomposition fumes). Additional experimental studies were also performed to determine the strength of energetic decomposition using Trauzl lead block tests.

## 2. Results and Discussion

### 2.1. Structure

**Figure 1:**
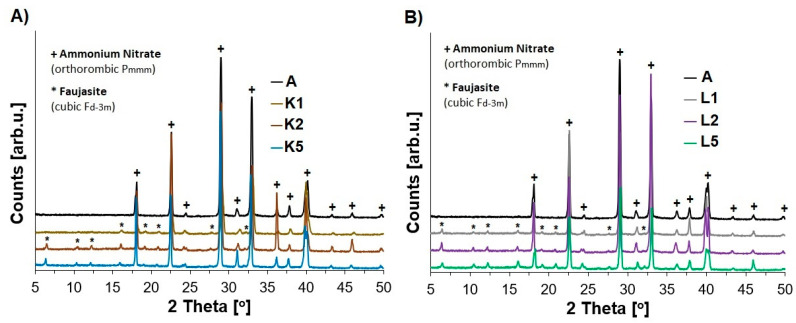
The impact of the addition of variously modified zeolite (1, 2, or 5% wt.) on the appearance of the XRD patterns of the studied ANFO samples: (**A**) ANFO samples modified with Cu_2_HFAU31 zeolite; (**B**) ANFO samples modified with Cu_5_NaFAU31.

We investigated the influence of the synthesis conditions of our ANFO materials on their crystallinity and structure (Figure 1). The analysis of the diffraction patterns confirmed the presence of an orthorhombic crystalline ammonium nitrate phase with the P_mmm_ symmetry (AN) [48,49]. Furthermore, it was indicated that the addition of even 1% wt. of faujasite (samples K1 and L1) resulted in the appearance of additional reflections assigned to the zeolite phase with the F_d-3m_ symmetry [50,51]. Higher loadings of zeolite in the prepared ANFO samples (exceeding 5% wt.—K2, K5, and K5 and L5) resulted in more apparent signals assigned to the zeolite additive. In our previous work [35], we also reported that the addition of 1 or 2% wt. of silica resulted in the appearance of a weak signal attributed to SiO_2_.

Analogous findings can be found in the research reported by Xu et al. [52], who investigated the addition of organic potassium salts to AN. They indicated that the application of this type of modifier could influence AN by the formation of hydrogen bonds by polar groups as a result of the interaction between ammonium and nitrate ions. Based on these considerations, we can expect that the blending of our Cu-faujasites (in the form of both Cu_2_HFAU31 and Cu_5_NaFAU31) with AN could result in structural changes in relation to the pure AN. However, no relevant differences were found in the appearance of XRD patterns of ANFO samples containing Cu-zeolite additive prepared from either protonic or sodium forms of faujasite.

**Figure 2:**
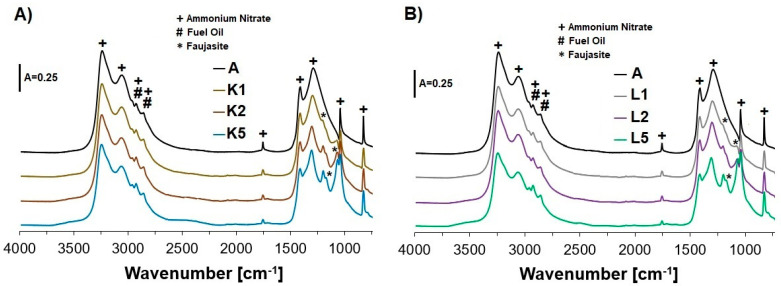
The impact of the addition of variously modified zeolite (1, 2, or 5% wt.) on the appearance of the FT-IR spectra of the studied ANFO samples: (**A**) ANFO samples modified with Cu_2_HFAU31 zeolite; (**B**) ANFO samples modified with Cu_5_NaFAU31.

The FT-IR spectra depicted for all samples in Figure 2 reflect the presence of the bands at the region of 3260–2850 cm^−1^, assigned to asymmetric vibrations of NH_4_^+^ in both stretching and deformation modes. In turn, the signals at 1755 cm^−1^ can be attributed to a stretching vibration or in-plane deformation of NO_3_^−^, or they may be due to a combination of an asymmetric deformation of ammonium cations with a lattice mode. Distinct maxima at 1410 and 1290 cm^−1^ could correspond to the triply degenerated deformation of NH_4_^+^ and the doubly degenerated stretching vibration of NO_3_^−^. Furthermore, the bands at 1040 and 825 cm^−1^ can be associated with in-plane symmetric stretching and out-of-plane deformation of NO_3_^−^, respectively [53]. The occurrence of the bands in the wavenumber region of ca. 2950–2850 cm^−1^ may also be due to -CH_2_- and -CH_3_ stretching vibrations from fuel oil [54]. 

Similar to the XRD results, the addition of even 1% wt. of Cu-containing faujasite (in the form of both Cu_2_HFAU31 (sample K1) and Cu_5_NaFAU31 (sample L1)) to AN in our ANFO-based samples led to the appearance of the bands attributed to the zeolite phase at ca. 1100–1200 cm^−1^. These bands may originate from asymmetric stretching vibrations inside TO_4_ tetrahedra (where T = Si or Al): ν_as_ (←OT→←O) or structurally sensitive asymmetric stretching vibrations between TO_4_ tetrahedra: ν_as_ (T←O→←T) [55]. Increasing the Cu-zeolite loading in the prepared ANFO-based samples (samples K2, K5, L2, and L5) caused more intense signals coming from the zeolite phase. Analysis of the FT-IR spectra of the studied ANFO-based samples led to the conclusion that the rest of the bands related to the zeolite phase (e.g., symmetric stretching ←TOT→ vibrations at 820–750 cm^−1^) overlapped with AN skeletal vibrations.

### 2.2. The Status of the Surface

The status of the surface of the synthesized ANFO samples should allow us to predict whether and how accurately we could blend ammonium nitrate with other ingredients, as well as how this factor could influence the energetic properties of ANFO-based materials. 

**Figure 3:**
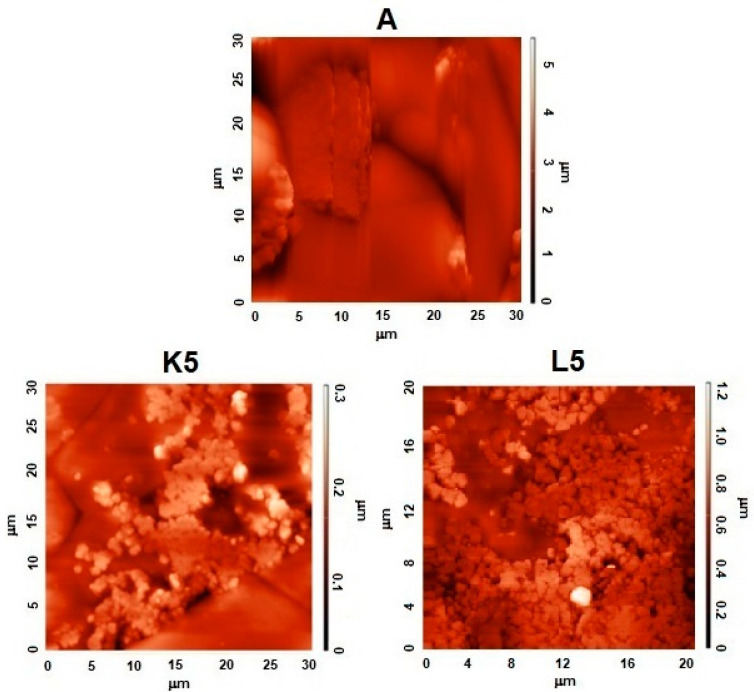
AFM images and visualization of the surface of ANFO samples containing 5% wt. of Cu-faujasite in the view from the top in the plane of the page.

**Figure 4:**
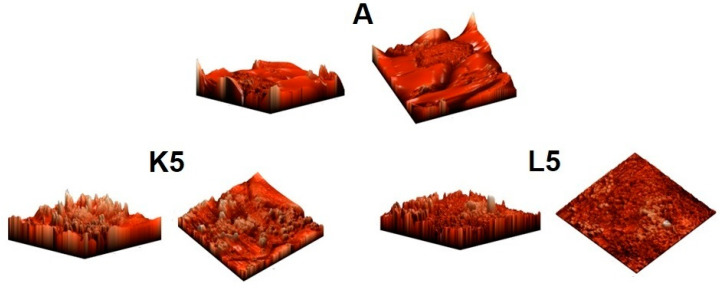
AFM visualization of the surface of ANFO samples in diagonal projections.

AFM analysis indicated the presence of numerous surface deformations on AN prills in all studied samples (Figure 3 and Figure 4). The presence of irregular-shaped AN grains was also apparent (diameters of 10–15 μm—see values on the axis on the bottom and left side of the AMF images). The surface of the tested samples was hilly and was characterized by the existence of bulges with a height reaching 5 μm (in the view from the top in the plane of the page). 

The addition of Cu-containing zeolite FAU31 (samples K5 and L5) led to significant changes in the surface of the studied ANFO samples. First of all, the appearance of spherical-shaped crystals with a diameter of ca. 1 μm was observed, which was undoubtedly due to the zeolite phase. 

The blending of ammonium nitrate with Cu-zeolite (in the case of both Cu_2_HFAU31 and Cu_5_NaFAU31) led to the apparent smoothing of the surface of the prepared samples, meaning a reduction in surface folding (see values on the axis on the right side of the AMF images). However, the state of the resulting surface of the ANFO sample clearly depended on the route of sample modification. For ANFO modified with Cu_2_HFAU31 zeolite (sample K5), the maximal height of the bulges was 0.3 μm, whereas in the case of the counterpart treated with Cu_5_NaFAU31 zeolite (sample L5), this value reached 1.2 μm. 

**Figure A1:**
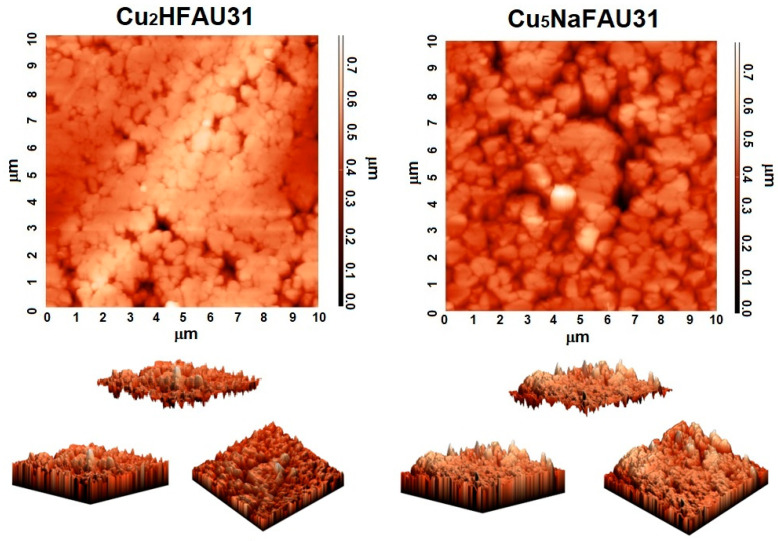
AFM images and visualization of the surface of Cu-faujasite additives in different projections.

The measured roughness of the surface of all prepared ANFO samples was expressed using the root-mean-square (RMS) topographical parameter, as defined in [56]. The RMS results are summarized in Table A1. At first sight, pure Cu-containing zeolite additives were characterized by much lower RMS in comparison with the reference ANFO sample (RMS = 1.297 μm). This fact can be explained by the much more uniform surface of faujasite (Figure A1) in relation to AN (Figure 3 and Figure 4). First of all, the variously modified Cu-faujasite zeolite Y grains were smaller than the AN prills. Interestingly, the state of the zeolite surface clearly depended on the route of zeolite modification. Cu_2_HFAU31 zeolite was characterized by a lower RMS value (0.088 μm) than the Cu_5_NaFAU31 analog (0.112 μm), which suggested that both the chemical form and the amount of copper in faujasite could influence the roughness of the zeolite samples. The blending of a pure ANFO sample (A) with Cu_2_HFAU31 or Cu_5_NaFAU31 (K- or L-series of ANFO-based samples) resulted in a reduction in the RMS value. It must be underlined that we observed smaller RSM values (lower roughness of the surface) for the ANFO-based samples belonging to the K series (0.050 μm < RMS < 0.228 μm) than for their counterparts from the L series (0.109 μm < RMS < 0.463 μm), which correlated strictly with the roughness of pure Cu-faujasite additives. Furthermore, increasing the loading of the zeolite additive caused a decrease in the roughness of the resulting ANFO samples, independent of the type of zeolite modifier applied (both Cu_2_HFAU31 and Cu_5_NaFAU31). Based on AFM images (Figure 3 and Figure 4) and RMS data (Table A1), we could conclude that the same zeolite could decrease roughness by itself, whereas the copper phase could cause the opposite effect. Our results are in line with the findings described in [57,58].

Brazdeikis et al. [57] investigated thin CuO films on MgO supports incorporated by molecular-beam epitaxy (MBE) using NO_2_ as an oxidant. The AFM images indicated changes in the surface status of the studied samples. The increasing thickness of the CuO on the MgO support led to an increase in the roughness of the CuO/MgO systems. Another effect was a random distribution of round-shaped CuO islands on the MgO surface [57]. Park et al. [58] studied Cu oxide thin films over Si wafers incorporated by radio frequency magnetron sputtering at various annealing temperatures. Park et al. [58] reported that both the Cu-phase grain sizes and the roughness of the prepared samples increased with the annealing temperature. However, the quantitative proportion between Cu_2_O, CuO, and Cu(OH)_2_ phases was independent of the annealing temperature. Hence, the roughness of the Cu phase deposited on the Si carrier depended on the Cu content and was simultaneously independent of the status of copper [58]. Therefore, in the case of our ANFO samples containing Cu-zeolite, the amount of Cu probably had a predominant impact on their roughness, whereas the chemical form of copper species had a lesser effect.

In our previous work [59], we applied Mg-containing faujasite as a modifier of ANFO. The zeolitic component was bare or contained Mg. Magnesium was incorporated via impregnation, ion exchange, or sonication methods, which resulted in the existence of magnesium in the form of MgO, or Mg^2+^, or MgO/Mg^2+^, respectively. We found that mixing ammonium nitrate with zeolite resulted in a noticeable reduction in the roughness of the surface of the ANFO samples; however, for each sample, the effect was different and independent of the chemical form of magnesium present in the zeolite. 

### 2.3. Thermal Analysis

**Figure 5:**
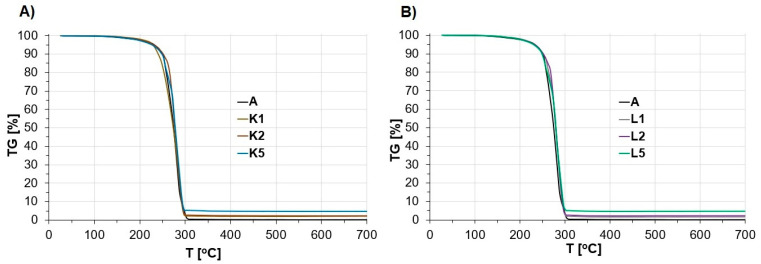
The appearance of TG curves of the studied ANFO samples faced with the addition of Cu-faujasite (1, 2, or 5% wt.): (**A**) ANFO samples modified with Cu_2_HFAU31 zeolite; (**B**) ANFO samples modified with Cu_5_NaFAU31.

**Figure 6:**
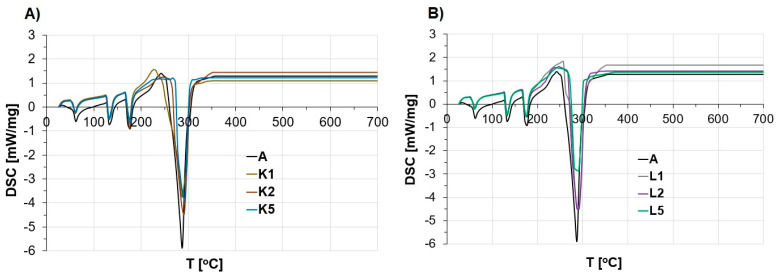
The appearance of DSC profiles of the studied ANFO samples faced with the addition of Cu-faujasite (1, 2, or 5% wt.): (**A**) ANFO samples modified with Cu_2_HFAU31 zeolite; (**B**) ANFO samples modified with Cu_5_NaFAU31.

The characterization of thermal properties was carried out to evaluate how the modification of the ANFO reference sample with various contents of Cu-containing FAU-type zeolite (in the form of both Cu_2_HFAU31 and Cu_5_NaFAU31) could influence the decomposition of ANFO. The TG curves and DSC profiles are given in Figure 5 and Figure 6, respectively. 

Based on TG data (Figure 5), we found that for all ANFO samples, a small mass loss found at a temperature below 250 °C was a result of the evaporation of water and FO from crystalline AN. The evaporation of liquid occurred initially in the surface pores, then from the liquid monolayer, and finally from further layers. However, according to Kwok and Jones [60], the distinction between FO evaporation from pores and monolayers is not well known. A separate issue is the wettability of AN, which determines its stability. The AN’s wettability depends strictly on several factors, e.g., porosity or the prill surface area [60]. 

Subsequent temperature growth up to 250–300 °C resulted in the decomposition of AN [61,62]. Independent of the route of the modification of ANFO with Cu-faujasite, we did not observe relevant differences in the appearance of the TG curves between the studied ANFO samples with the addition of zeolite at specific contents. However, the mass of the sample residue corresponded strictly with the weight content of the zeolite in the prepared ANFO samples. Specifically, for the ANFO reference sample (A), the sample mass residue was zero, whereas in the case of ANFO-based energetic materials containing Cu-zeolite, the mass residues were 1% (for samples K1 and L1), 2% (for samples K2 and L2), and 5% (for samples K5 and L5). However, it must be pointed out that the type of Cu-faujasite modifier (Cu_2_HFAU31 vs. Cu_5_NaFAU31) had no impact on the TG data. 

In our previous research, we performed TG analysis for pure FAU-type zeolite additives with a low Si/Al ratio [59]. We found that the observed mass loss in ANFO-based samples containing zeolite could also result from the desorption of water from the zeolite surface and internal parts of the zeolite grains [63].

Based on the DSC profiles (Figure 6), four endothermic peaks were found at 60, 135, 175, and ca. 290 °C. The first two signals were due to the crystallographic transformation of AN (from rhombic to trigonal, and from trigonal to regular, respectively) [53]. A third endothermic signal at ca. 175 °C corresponded to the melting point of AN. In turn, the occurrence of the fourth DSC peak, with a maximum of 287–293 °C, was strictly associated with the thermal decomposition of AN [64]. Analysis of the appearance of the DSC profiles led to the conclusion that the type of zeolite additive (Cu_2_HFAU31 vs. Cu_5_NaFAU31) and its content (1% wt. vs. 2% wt. vs. 5% wt.) influenced the thermal effect resulting from the decomposition of such prepared Cu-zeolite-containing ANFO-based energetic materials. The most significant effect was observed for the ANFO modified with 5% wt. of Cu_5_NaFAU31 zeolite containing mainly copper oxides (L5), for which the thermal DSC signal was −2.85 mW/mg. In contrast, the weakest changes were documented for the counterparts including 1% wt. or 2% wt. of zeolite additive, regardless of the chemical form of copper existing in the zeolite additive, whose DSC peak value was ca. −4.5 mW/mg. In the case of the ANFO sample containing 5% wt. of Cu_2_HFAU31 zeolite with a cationic Cu form (K5), we observed a medium DSC effect (−3.9 mW/mg). For comparison, for the ANFO reference sample (A), the DSC peak was −5.8 mW/mg (pure ANFO). Nevertheless, neither the type of zeolite additive nor its content in the resulting ANFO samples distinctly influenced the temperature of the DSC signals. 

Furthermore, it must be emphasized that despite the exothermic character of AN’s decomposition, we observed endothermic DSC peaks due to measurements conducted in open crucibles under aerobic conditions. This action prevented a sharp increase in temperature and pressure during experiments in the tested system, which guaranteed safe measurements.

Our results are in line with those of our previous research [59], in which we reported that the application of Mg-containing zeolite Y (faujasite of Si/Al of 2.65) decreased the energy required for the decomposition of ammonium nitrate. The scale of the thermal effects depended on the method of introducing magnesium into zeolite. For ANFO samples containing Mg-Y prepared via impregnation, ion exchange, and sonication techniques, the thermal DSC effects were −4.32 mW/mg, −4.20 mW/mg, and −5.02 mW/mg, respectively. A direct comparison of the current results (Cu-containing FAU31 additives) with Mg-Y analogs reported in [59] revealed that Cu-zeolites turned out to be more efficient. 

Thermal effects related to the decomposition of AN can also depend on the grain size of the modifier. In our previous paper [65], we reported that when we used microstructured charcoal (MC) as a modifier of ANFO, smaller MC grains reduced the ANFO decomposition temperature (from 292 to 272 °C). In the case of zeolites, we did not need to study the impact of the size of the zeolite grains, because we used the zeolites as powders. Another factor determining the thermal properties of the prepared ANFO samples is the provenance of AN [66]. We also found that the AN porous prills used in the mining industry undergo decomposition at a lower temperature than their AN counterparts used in agriculture as fertilizers. 

In the case of ANFO, the addition of fuel oil to ammonium nitrate may lead to complex reactions, in which nitrate ions could act as an oxidizing agent, whereas ammonium ions could be responsible for the reduction reactions [62]. Other exemplary fuels, such as paper or wood, could cause the autoignition of fuel components at ca 250 °C. Therefore, it could be expected that the reactivity of the fuel component should not occur below 250 °C [62,67]. On the other hand, Fedoroff et al. [68] reported that ammonium nitrate decomposed above 230 °C, but could deflagrate at temperatures higher than 325 °C.

### 2.4. Energetic Performance

The results of the thermodynamic calculations—including, among others, the pressure, temperature, heat, velocity of energetic decomposition, and fume composition from 1 kg of energetic material—are summarized in Table 1 and Table 2. In the conducted calculations of the energetic properties of the prepared materials, we applied the Chapman–Jouguet (C-J) decomposition theory, which assumes instantaneous chemical reactions and thermodynamic equilibrium of the decomposition products [69]. 

From the data given in Table 2, it may be concluded that calculated energetic parameters like pressure, temperature, heat, and velocity of decomposition, as defined in [70], increased with the Cu-zeolite content in the prepared ANFO samples. The observed effects were similar for the ANFO samples containing Cu-faujasite, regardless of the type of zeolite modifier—Cu_2_HFAU31 (K series) or Cu_5_NaFAU31 (L series)—although it seemed that the calculated energetic parameters were somewhat higher for the K series than for L analogs. 

For the addition of 1, 2, or 5% wt. of zeolite to the reference ANFO sample, the decomposition pressure rose from 3838 MPa to 4022 MPa or 4014 MPa, 4166 MPa or 4118 MPa, and 4419 MPa or 4422 MPa for the K1 or L1, K2 or L2, and K5 or L5 ANFO samples, respectively. In turn, the decomposition temperature increased from 2970 K (for sample A) up to 3200 K vs. 3193 K, 3405 K vs. 3386 K, and 3939 K vs. 3914 K for the K1 or L1, K2 or L2, and K5 or L5 ANFO samples, respectively. Similar trends were observed for the heat of energetic decomposition, for which the presence of zeolite in the resulting ANFO-based materials caused the growth of this energetic parameter from 3913 kJ/kg (for sample A) to 4363 kJ/kg vs. 4348 kJ/kg (K1 vs. L1), 4766 kJ/kg vs. 4738 kJ/kg (K2 vs. L2), or 5872 kJ/kg vs. 5804 kJ/kg (K5 vs. L5).

The results of the thermodynamic calculations were in strong accordance with the research reported in [33], where we found that the application of metallic dust to modify ANFO caused a significant increase in the heat of energetic decomposition from 3940 kJ/kg (for the ANFO reference sample) to 4510 J/kg (for ANFO + Al), 4500 kJ/kg (for ANFO + Mg), or 4000 kJ/kg (for ANFO + Cu) due to the metallic powder acting as a fuel, which was a spare source of energy [29,30,31,32,33]. On the other hand, the elevated values of the energetic parameters could have originated from the afterburning effect, which might have resulted from the secondary reaction between unreacted FO (and/or partially oxidized post-decomposition fumes formed during primary reactions) and the surrounding air [71,72]. 

A search for previous research on ANFO-based energetic materials modified with zeolites revealed it to be virtually non-existent. Only in [59] did we report research on the application of variously modified Mg-containing zeolite Y as a modifier of ANFO-based energetic materials. We also studied whether the route via which Mg was introduced into the zeolite skeleton (impregnation vs. ion exchange vs. ultrasonic irradiation) could have any impact on the usefulness of such prepared zeolite Y as an enhancer of ANFO-based energetic materials. First of all, we found that the addition of zeolite Y enhanced the energetic performance of such prepared ANFO via increases in the pressure, temperature, and heat of the decomposition (up to 8%, 16%, and 23%, respectively) in comparison with the zeolite-free ANFO samples. 

**Figure 7:**
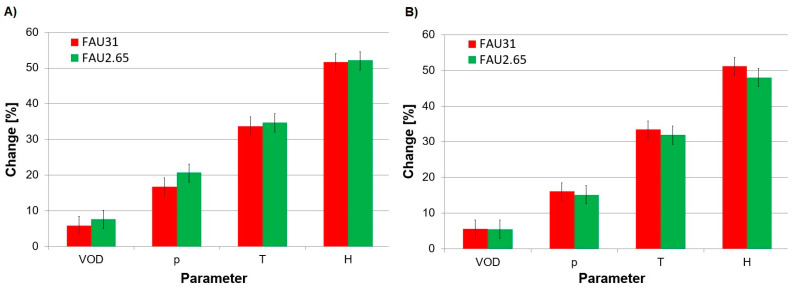
The influence of humidity and Si/Al ratio in zeolite additives on the energetic properties of the prepared ANFO samples: (**A**) anhydrous conditions; (**B**) hydrated samples. Energetic parameters obtained for the reference sample (designated as “A”) are given in Table 2.

In a separate step, we also investigated the impact of the Si/Al ratio in zeolite additives on the energetic properties of the prepared ANFO samples (Figure 7). Simply put, we performed research on Cu-free parent FAU-type zeolites. A direct comparison of the results from thermodynamic calculations of energetic parameters in anhydrous conditions indicated that zeolite additives with a lower Si/Al ratio could improve the energetic performance of ANFO samples compared to their counterparts with a Si/Al ratio of 31 (Figure 7A). The obtained results could be explained by the fact that zeolite with a lower Si/Al ratio automatically contains a higher aluminum content, playing the role of fuel as a combustible component of this type of energetic material [29,30,31,32,33].

We observed a reversed effect under hydrated conditions (Figure 7B). In the presence of humidity in the studied systems, the addition of zeolite with a higher Si/Al value revealed enhanced energetic properties compared to ANFO samples containing zeolite counterparts with a lower Si/Al ratio. The obtained results of thermodynamic calculations could be explained by the fact that zeolite with a higher aluminum content (lower Si/Al ratio) is more hydrophilic [73]. Hence, an elevated moisture content in the ANFO samples could reduce the energetic parameters [74,75,76]. 

Direct comparison of the results concerning energetic behavior obtained in the current research with those reported in [59] leads to the conclusion that ANFO energetic materials doped with Cu-faujasite with a Si/Al ratio of 31 are characterized by more satisfactory energetic performance than their counterparts modified with Mg-Y with a Si/Al ratio of 2.65, regardless of the preparation route. The observed differences in energetic properties between ANFO-based energetic materials modified with two kinds of faujasites could originate mainly from their various Si/Al ratios. It must be pointed out that, under real experimental conditions, the existence of humidity is unlikely to be excluded, due to the relatively high hydrophilicity of both zeolite additives and AN by themselves [2,53,73]. 

Concerning the velocity of decomposition (VOD), the introduction of 1, 2, or 5% wt. of zeolite into pure ANFO (sample A) resulted in the growth of this parameter from 4392 m/s to 4460 m/s, 4514 m/s, or 4629 m/s for K1, K2, or K5, as well as to 4456 m/s, 4507 m/s, or 4616 m/s for L1, L2, or L5 ANFO samples, respectively (Table 1).

Our findings are in line with the results of the VOD values with respect to the correlation with the modifier content [35], as well as with the heat values, which were linear [77]. In our previous research [35], we found that increasing the silica content in ANFO led to ambiguous changes in the VOD values. Similarly to silica, zeolite can be considered to be a source of oxygen (oxygen-bearing component), as well as supporting aluminum as a fuel for ANFO. These observations were also confirmed by Miyake et al. [78], who studied the velocity of the energetic decomposition of materials based on AN mixed with activated carbon (AC). It was found that the VOD increased with the AC content. Similar conclusions were postulated in the case of ANFO mixed with microstructured charcoal (ANFO + MC), where the addition of MC to the ANFO caused an increase in the VOD with the amount of MC [65].

We also performed thermodynamic calculations referring to the productivity of post-decomposition fumes (among others, water vapor, N_2_, CO_x_, and NO_x_, expressed in m^3^) as a result of the decomposition of 1 kg of ANFO-based energetic materials. From the data listed in Table 2, it may be concluded that increasing the zeolite content in the resulting ANFO samples led to a reduction in the total emission of post-decomposition fumes from 1057 m^3^/kg (for the reference ANFO sample) to 1041 m^3^/kg, 1027 m^3^/kg, or 990 m^3^/kg for the ANFO samples containing 1, 2, or 5% wt. of Cu-faujasite, respectively. Interestingly, we observed the same results independent of the kind of Cu-zeolite additive (Cu_2_HFAU31 vs. Cu_5_NaFAU31 for the K or L series, respectively). Hence, it could be postulated that neither the chemical form of copper nor its content in the zeolite influenced the production of post-decomposition fumes from ANFO samples modified with Cu-faujasite. 

The reduction in the total emission of post-decomposition fumes as a result of the application of the additives in ANFO-based energetic materials was confirmed by Maranda et al. [30,31], who reported that increasing the Al powder content caused a relevant reduction in the toxic fumes formed during ANFO’s decomposition. Also, in our previous studies on the application of MC [65,79] and SiO_2_ [35], we found from thermodynamic calculations that increasing the modifier content caused a reduction in the total emission of post-decomposition fumes.

The calculated reduction in the emission of post-decomposition fumes as a result of the simulated decomposition of ANFO-based energetic materials was mainly due to the decrease in the production of water vapor and nitrogen, as well as a lesser content of CO_x_. Surprisingly, the introduction of Cu-faujasite as an ANFO additive caused a slight growth in the release of NO_x_ among the post-decomposition fumes, as well as other products (Table 2). It seems that Cu-faujasites revealed different behavior than expected for Cu-zeolite catalysts [37,38,39,40,41,42,43,44,45], for which the removal of NO_x_ took place. Therefore, it may be postulated that our Cu-containing zeolites with an FAU structure type (as both Cu_2_HFAU31 and Cu_5_NaFAU31) play another role than catalysts, and further investigations explaining the impact of Cu-zeolites on the mechanism of ANFO’s decomposition should be carried out in the next step of our research. Nevertheless, elevated emissions of NO_x_ could result from the increasing values of oxygen balance for zeolite-containing ANFO energetic materials in comparison with the zeolite-free ANFO sample. On the other hand, the calculated changes were not as high. 

The relationship between the production of oxides among post-decomposition fumes and oxygen balance was confirmed by the results reported both in our previous research [35,65] and by other scientists, including Bhattacharyya et al. [80], Sapko et al. [81], Mainiero et al. [82], Onederra et al. [83], Chaiken et al. [84], and Rowland et al. [85]. 

Last, but not least, we performed experiments on the strength of the decomposition of prepared ANFO-based energetic materials containing variously modified Cu-faujasite using the Trauzl lead block test (Table 3). From the comparison of the differences between the initial volume of the lead blocks and their volume after the energetic decomposition, it may be concluded that the addition of zeolite additives generally enhanced the expansion of the lead blocks as a result of the decomposition of the studied energetic materials. Specifically, the addition of 1, 2, or 5% wt. of Cu_2_HFAU31 zeolite into the reference ANFO sample (A) caused the lead block’s volume to increase from 231.2 cm^3^ to 260.0 cm^3^, 295.5 cm^3^, or 256.4 cm^3^ for K1, K2, or K5. In the case of the L series, we observed weaker effects. For the L1 and L5 samples, the addition of Cu_5_NaFAU31 zeolite led to an increase in the expansion of the lead block to 271.6 cm^3^ and 253.8 cm^3^, respectively. An opposite effect was found for the L2 sample, for which we observed a smaller expansion of the lead black (223.2 cm^3^) compared with the reference ANFO sample. Generally, no distinct correlation was found between the Cu-zeolite content in the ANFO samples and their energetic performance. However, it can be postulated that the ANFO samples modified with Cu_2_HFAU31 (belonging to the K series) revealed more visible growth in the expansion of the lead block’s volume, reflecting a higher strength of the energetic decomposition.

Our results from the Trauzl lead block test can be compared with the results reported by Kramarczyk et al. [32], who investigated the effects of the addition of aluminum to emulsion bulk-type energetic materials on their properties. It was found that the growth of the lead blocks as a result of the energetic decomposition of the samples was correlated strongly with the aluminum content. The net expansion values ranged from 5.0 to 22.5 cm^3^ per 2% wt. of added Al. The maximum volume was nearly 360 cm^3^ for the sample containing 7% wt. of Al, which corresponded to an increase of ca. 20% in comparison with the Al-free reference sample.

Generally, from the analysis of the energetic performance of the prepared ANFO samples, it may be concluded that the enhancement of their energetic properties caused by the addition of Cu-containing zeolite with an FAU-type structure to ANFO was correlated distinctly with the status of their surface and a lesser content with their structure and thermal properties. The observed enhancement of the energetic parameters increased with the Cu-faujasite content in the studied ANFO samples and was higher for zeolite counterparts containing copper in monovalent cationic exchange positions (K series modified with Cu_2_HFAU31) than for the ANFO samples containing Cu_5_NaFAU31 (belonging to the L series). 

## 3. Materials and Methods

### 3.1. Materials and Sample Preparation

Porous prilled ammonium nitrate (UltrAN 70) with a purity of 99.4% and containing ca. 34.5% nitrogen was supplied by Yara International ASA (Oslo, Norway). UltrAN 70 was characterized by a prill diameter of 1.0–2.0 mm and bulk density in the range of 670–720 kg/m^3^. The moisture did not exceed 0.3% wt. In turn, fuel oil (FO) was supplied by Silesia Oil Sp. z o.o. and consisted of C_10_–C_20_ hydrocarbons. The detailed characterization of FO was reported in [86]. The ANFO reference sample (denoted as “A”) was prepared via simple mixing of UltrAN 70 with FO at a weight ratio of 94.0:6.0 (Table 1), which led to reaching almost zero oxygen balance, i.e., no excess or deficiency of oxygen in the balance of the composition of the energetic material. 

The zeolite with an FAU-type structure of Si/Al of 31 was supplied by Zeolyst Company (Farmsum, The Netherlands). The commercial zeolite (CBV760) was in the protonic form (HFAU31). The commercial zeolite was modified with copper. The introduction of Cu into faujasite was conducted via a wet impregnation procedure. The Cu_2_HFAU31 zeolite contained 2% wt. of Cu and was based on the HFAU31 zeolite. Meanwhile, the Cu_5_NaFAU31 zeolite contained 5% wt. of Cu and was produced as a result of the impregnation of the sodium form of commercial faujasite (NaFAU31) with copper. A detailed description of the production of Cu-containing FAU31 zeolites is given in the Appendix A (Table A2). We chose the Cu_2_HFAU31 and Cu_5_NaFAU31 zeolite samples as the additives for ANFO samples because we reported the highest differentiation concerning the status of copper for those two samples [46]. 

The Cu_2_HFAU31 and Cu_5_NaFAU31 zeolite samples acted as modifiers of the ANFO samples’ physicochemical properties and energetic behavior. The zeolite samples were added to the ANFO via simple blending. Depending on the weight content of the zeolite in the resulting ANFO-type material (1, 2, or 5% wt.), as well as the type of the added zeolite (“K” for Cu_2_HFAU31 vs. “L” for Cu_5_NaFAU31), the ANFO samples were denoted as K1 vs. L1, K2 vs. L2, or K5 vs. L5 (Table 4).

### 3.2. Characterization Methods

We investigated the influence of the synthesis conditions of our ANFO materials on their crystallinity and structure. The crystallinity of the ANFO samples was determined by the X-ray diffraction (XRD) technique using a PANalytical X’Pert PRO MPD diffractometer equipped with a CuKα generator (λ = 1.5418 Å, 40 kV, 30 mA). The XRD measurements were performed at 2θ angles in the range of 5–50°, with 0.033° per step.

Structural analysis of the prepared ANFO samples was conducted using a Nicolet iS10 spectrometer (Thermo Scientific, Waltham, MA, USA). The spectrometer was equipped with an MCT detector in the attenuated total reflectance (ATR) mode. The FT-IR analysis was carried out at 4000–650 cm^−1^, with a resolution of 4 cm^−1^. The number of scans during a single measurement was 64.

The status of the surface of our samples allowed us to predict whether and how accurately we could blend ammonium nitrate with other ingredients, as well as how this factor could influence the decomposition properties of ANFO. The status of the surface of the prepared ANFO samples was determined using an NT-MDT Solver BIO atomic force microscope (AFM) equipped with the SMENA SFC050L scanning head. All experiments were performed in air and in semi-contact mode using high-resolution silicon probes (NT-MDT ETALON probes, HA NC series, polysilicon cantilevers with resonance frequencies 140 kHz +/− 10% or 240 kHz +/− 10% and force constants 4.4 N/m +/− 20% or 9.5 N/m +/− 20%, respectively; a typical curvature radius of the tip was 10 nm and cone angle was less than 20°). The received images were associated with the randomly chosen areas over the substrate and within the scan area suitable for the investigated samples. The root-mean-square (RMS) topographical parameter defined the roughness of the particle layers and was determined by the ex situ AFM method and calculated using Gwyddion 2.56 software. A detailed description of the RMS method can be found in [56]. 

To investigate the influence of the addition of the variously modified zeolite Y on the thermal decomposition of ANFO, thermal analysis was conducted. Thermal analysis of the prepared ANFO samples, consisting of thermogravimetry (TG) and differential scanning calorimetry (DSC), was conducted using the NETZSCH STA 409 PC/PG instrument. All measurements were conducted at 20−700 °C, with a temperature ramp of 10 °C/min. All studied ANFO samples were exposed to airflow (30 mL/min) in both the furnace and the balance chamber. Air acted as a stimulator of decomposition conditions. For all experiments, the studied ANFO sample (20 mg) was located in the DSC aluminum pan. To obtain the correct TG baseline, the same heating profiles were used for both the empty pan and the pan containing a measured sample. The TG drift was ca. 5 μg, which corresponded to 0.02 mass%.

To determine the energetic properties of the prepared ANFO samples, we performed thermodynamic calculations. Theoretical considerations were carried out using EXPLO5 software (version v5.05, OZM Research S.R.O., Hrochův Týnec, Czech Republic) with a calculation error of 5%. Thermodynamic calculations were based on the Chapman–Jouguet and Becker–Kistiakowsky–Wilson models [70] and referred to chosen energetic parameters like pressure, temperature, heat, velocity, and the total volume of post-decomposition fumes that resulted from 1 kg of ANFO-based energetic material. 

Trauzl lead block tests were performed to reflect the strength of the energetic decomposition. These measurements were based on determining the expansion capacity produced by the decomposition of 10 g of energetic material in a cylindrical lead block with a diameter of 200 mm and height of 200 mm. The resulting expansion was compared to that produced with 10 g of picric acid, which played the role of a reference charge. The initial volume of a lead block was compared to the expansion produced by the studied energetic material. A detailed description of the test procedure was reported in [87].

## 4. Summary and Conclusions

In the present study, we investigated Cu-containing FAU-type zeolite as a modifier of eco-friendly ANFO-based energetic materials. All studied physicochemical properties (including crystallinity, structure, surface, thermal analysis, and energetic performance) of the prepared zeolite-containing ANFO-based materials were compared with the zeolite-free counterpart (acting as a reference sample). 

Analysis of either X-ray diffraction (XRD) patterns or infrared (IR) spectra revealed the appearance of additional reflections or bands assigned to the zeolite phase as a result of the addition of the Cu-faujasite to the reference ANFO sample. 

Analogous conclusions could be drawn based on the thermal analysis, which indicated that the addition of Cu-FAU zeolite resulted in an apparent reduction in the energy required for the decomposition of the ANFO-based energetic material. The effect was the most significant for the ANFO modified with 5% wt. of faujasite containing copper mainly in the oxide form. 

Analysis of atomic force microscopy (AFM) images indicated the presence of numerous surface deformations on AN prills in all studied samples, as well as the occurrence of irregular-shaped AN grains. The blending of ammonium nitrate with Cu-faujasite led to the apparent smoothing of the surface of the prepared samples, which increased with the zeolite content in the studied ANFO samples and was particularly apparent for FAU-type zeolite containing a lower amount of copper. 

Thermodynamic calculations indicated that the addition of Cu-containing zeolite with an FAU-type structure to ANFO led to the growth of energetic decomposition parameters like pressure, temperature, heat, and velocity and resulted in a relevant reduction in the total emission of post-decomposition fumes, which is strongly desirable from an ecological point of view. Similar conclusions could be drawn from the analysis of the Trauzl lead block tests, where the addition of Cu-faujasite to ANFO caused an increase in the strength of the energetic decomposition. Both the calculated and measured energetic behavior of the prepared ANFO-based samples correlated strictly with the status of their surface and, to a lesser extent, with their structure and thermal properties. The observed enhancement of the energetic properties increased with the Cu-faujasite content in the studied ANFO samples and was higher for zeolite counterparts containing copper in monovalent cationic exchange positions. 

In the case of Cu-free zeolite with an FAU-type structure, we investigated the impact of the Si/Al ratio in zeolite additives on the energetic performance of the prepared ANFO samples. It was found that, under anhydrous conditions, zeolite additives with a lower Si/Al ratio were better enhancers of the energetic properties of ANFO-based materials compared to their counterparts with a higher Si/Al ratio. This could be explained by the fact that zeolites with a lower Si/Al ratio contained more aluminum, which acted as a fuel. In the case of hydrated conditions, we observed the reverse effect due to the higher hydrophilicity of zeolites with a low Si/Al ratio, which reduced the energetic parameters. 

It must be pointed out that both the energetic performance and the analysis of the post-decomposition fumes indicated that our ANFO-based materials could be used as low-emission sources of energy for further industrial and individual purposes. 

The obtained results of both experimental research and thermodynamic calculations on the application of zeolites as modifiers of energetic materials, apart from answers, generated even more questions, which constitute the foundation of further considerations and, thus, open the door for further scientific plans related to this topic. One example could be knowledge of the influence of the addition of variously modified zeolites to ANFO on the decomposition mechanism of this kind of energetic material. The designed thermodynamic calculations allow for determining the interactions of each element present in the zeolite with the AN and FO phases. Furthermore, the use of simulations also allows for comparing the experimental and theoretical values to determine the energetic properties of the tested ANFO-based materials.

## Figures and Tables

**Table 1:** Selected calculated energetic properties of ANFO samples with the addition of variously modified zeolite FAU31.

Sample	Decomposition Pressure [MPa]	Decomposition Temperature [K]	Heat of Decomposition [kJ/kg]	VOD [m/s]
A	3838	2970	3913	4392
K1	4022	3200	4363	4460
K2	4166	3405	4766	4514
K5	4419	3939	5872	4629
L1	4014	3193	4348	4456
L2	4118	3386	4738	4507
L5	4422	3914	5804	4616

**Table 2:** Calculated volumes of the selected decomposition fumes for ANFO samples with the addition of variously modified zeolite FAU31.

Sample	N_2_ [dm^3^/kg]	NO_x_ [dm^3^/kg]	CO_x_ [dm^3^/kg]	H_2_O [dm^3^/kg]	Other [dm^3^/kg]	Total Post-Decomposition Fumes [dm^3^/kg]	Oxygen Balance [%]
A	287	1	107	655	7	1057	−0.99
K1	274	4	106	647	10	1041	−0.99
K2	260	7	104	637	19	1027	−0.98
K5	220	19	99	604	48	990	−0.96
L1	274	3	106	647	11	1041	−0.99
L2	261	7	104	637	18	1027	−0.98
L5	222	19	100	605	44	990	−0.96

**Table 3:** Results of Trauzl tests.

Sample	Initial Volume [ml]	Final Volume [ml]	Difference [ml]	Net Expansion in a Lead Block [cm^3^]
Picric acid	65.5	381.2	315.7	-
A	67.6	302.8	235.2	231.2
K1	67.7	325.9	258.2	260.0
K2	64.8	358.2	293.4	295.5
K5	65.5	320.1	254.6	256.4
L1	67.0	322.5	255.5	271.6
L2	66.9	276.8	209.9	223.2
L5	65.8	304.5	238.7	253.8

**Table 4:** Chemical composition of energetic materials and their synthesis conditions; the total sample mass was normalized to 5.00 g.

Sample	Chemical Composition [% wt.]			Description
	Ammonium Nitrate	Fuel Oil	Zeolite FAU	
A	94.00	6.00	0.00	Commercial ANFO, reference sample (5.00 g)
K1	93.06	5.94	1.00	ANFO (4.95 g) + Cu_2_HFAU31 (0.05 g). Zeolite HFAU31 containing 2% wt. of Cu introduced via impregnation method
K2	92.12	5.88	2.00	ANFO (4.90 g) + Cu_2_HFAU31 (0.10 g). Zeolite HFAU31 containing 2% wt. of Cu introduced via impregnation method.
K5	89.30	5.70	5.00	ANFO (4.75 g) + Cu_2_HFAU31 (0.25 g). Zeolite HFAU31 containing 2% wt. of Cu introduced via impregnation method.
L1	93.06	5.94	1.00	ANFO (4.95 g) + Cu_5_NaFAU31 (0.05 g). Zeolite NaFAU31 containing 5% wt. of Cu introduced via impregnation method.
L2	92.12	5.88	2.00	ANFO (4.90 g) + Cu_5_NaFAU31 (0.10 g). Zeolite NaFAU31 containing 5% wt. of Cu introduced via impregnation method.
L5	89.30	5.70	5.00	ANFO (4.75 g) + Cu_5_NaFAU31 (0.25 g). Zeolite NaFAU31 containing 5% wt. of Cu introduced via impregnation method.

## Data Availability

The data presented in this study are available upon request from the corresponding author Łukasz Kuterasiński.

## Appendix A

**Table A1:** The roughness of the surface of the studied ANFO samples, determined using the root-mean-square parameter from the AFM technique.

Sample	RMS [μm]
A	1.297
K1	0.228
K2	0.152
K5	0.050
L1	0.463
L2	0.109
L5	0.120
Cu_2_HFAU31	0.088
Cu_5_NaFAU31	0.112

**Table A2:** Preparation and specification of zeolite-based additives.

Sample	Modification	Remarks
HFAU31	Commercial zeolite of FAU-type structure (Si/Al = 31) was produced by Zeolyst Company (CBV 760). It was dealuminated by steaming and acid treatment by the producer.	Protonic form
Cu_2_HFAU31	HFAU31 was impregnated with Cu(NO_3_)_2_.	2%wt. of Cu in zeolite; much Cu^+^_exch._ and H^+^_,_ little Cu^+^_ox._ and Cu^2+^
NaFAU31	HFAU31 was 5-fold ion-exchanged with 0.5 M NaNO_3_ at 80 °C for 2 h.	Sodium form
Cu_5_NaFAU31	NaFAU31 impregnated with Cu(NO_3_)_2_.	5%wt. of Cu in zeolite; little Cu^+^_exch._ and H^+^, much Cu^2+^, Cu^+^_ox_
